# Effects of Groove Sealing of the Posterior Occlusal Surface and Offset of the Internal Surface on the Internal Fit and Accuracy of Implant Placements Using 3D-Printed Surgical Guides: An In Vitro Study

**DOI:** 10.3390/polym13081236

**Published:** 2021-04-11

**Authors:** Jung-Hwa Lim, Enkhjargal Bayarsaikhan, Seung-Ho Shin, Na-Eun Nam, June-Sung Shim, Jong-Eun Kim

**Affiliations:** 1 Department of Prosthodontics, Oral Research Science Center, BK21 FOUR Project, Yonsei University College of Dentistry, Yonsei-ro 50-1, Seodaemun-gu, Seoul 03722, Korea; erin850313@gmail.com (J.-H.L.); shin506@prostholabs.com (S.-H.S.); jennynam90@yuhs.ac (N.-E.N.); 2 Department of Prosthodontics, Yonsei University College of Dentistry, Yonsei-ro 50-1, Seodaemun-gu, Seoul 03722, Korea; ejbayar@gmail.com (E.B.); jfshim@yuhs.ac (J.-S.S.)

**Keywords:** 3D-printed surgical guide, CAD/CAM, offset, groove sealing, internal fit, implant placement, coverage, accuracy

## Abstract

This study evaluated the internal fit and the accuracy of the implant placement position in order to determine how the surface shape of the tooth and the offset influence the accuracy of the surgical guide. The acquired digital data were analyzed in three dimensions using 3D inspection software. The obtained results confirmed that the internal fit was better in the groove sealing (GS) group (164.45 ± 28.34 μm) than the original shape (OS) group (204.07 ± 44.60 μm) (*p* < 0.001), and for an offset of 100 μm (157.50 ± 17.26 μm) than for offsets of 30 μm (206.48 ± 39.12 μm) and 60 μm (188.82 ± 48.77 μm) (*p* < 0.001). The accuracy of implant placement was better in the GS than OS group in terms of the entry (OS, 0.229 ± 0.092 mm; GS, 0.169 ± 0.061 mm; *p* < 0.001), apex (OS, 0.324 ± 0.149 mm; GS, 0.230 ± 0.124 mm; *p* < 0.001), and depth (OS, 0.041 ± 0.027 mm; GS, 0.025 ± 0.022 mm; *p* < 0.001). In addition, the entries (30 μm, 0.215 ± 0.044 mm; 60 μm, 0.172 ± 0.049 mm; 100 μm, 0.119 ± 0.050 mm; *p* < 0.001) were only affected by the amount of offset. These findings indicate that the accuracy of a surgical guide can be improved by directly sealing the groove of the tooth before manufacturing the surgical guide or setting the offset during the design process.

## 1. Introduction

The recent advent of cone beam computed tomography (CBCT), computer-aided design (CAD), and computer-aided manufacturing (CAM) technologies has played a major role in the advancement of the clinical workflow related to implant surgery and the manufacturing process of implant prostheses [1]. In particular, the advancements in implant planning software have enabled the development of restoration-driven implantology, a concept to achieve long-term functional and aesthetic success through comprehensive diagnosis and appropriate treatment planning with the help of static guided implant surgery [2].

Additive manufacturing (AM) is the process of joining materials to make parts from three-dimensional (3D) model data, usually layer upon layer [3], which enables complex or customized designs [4,5]. AM has been adopted in dentistry for the fabrication of surgical guides, dental models, provisional restorations, and custom trays. In addition, the recent introduction of relatively inexpensive in-office stereolithographic 3D printers has further popularized AM in dentistry, particularly with the use of vat polymerization [6,7]. In clinical applications, after acquiring 3D image data using CBCT or scanners (facial or intraoral), it is possible to manufacture 3D objects that require customization [4,8,9].

A surgical guide can be used to accurately transfer the planned implant position into the oral cavity and, consequently, a more accurate implant placement can be achieved by static guided implant surgery in comparison to freehand surgery [10,11,12,13]. Moreover, data from a randomized controlled trial with a five-year follow-up demonstrated that post-surgical pain and the amount of marginal bone loss in a computer-guided group was lower when compared with a freehand group [14]. The sleeve is part of a surgical guide, of a precise diameter, that allows the drill to accurately and stably move along the insertion path and optimize the direction and depth of the implant drill [15]. The effect of a sleeve is best expressed by limiting the movements of the drill, which can be strictly controlled by ensuring a minimum clearance between the sleeve and the drill [15].

The surgical guide can be supported by teeth, mucosa, or bone. Previous studies found that tooth-supported surgical guides were more accurate than those supported by tissue and bone [16]. It has further been demonstrated that the number, location, and anatomy of the teeth supporting the surgical guide can also impact its precision, and including posterior teeth in the support area can improve the stability of the surgical guide [17,18].

One limitation of a surgical guide is that its internal surface cannot perfectly fit the anatomy of the teeth on which it is placed. Errors due to 3D printing or minor errors on the internal surface of the surgical guide can cause premature contact with the occlusal surface. The occlusal surface consists of fine and complex anatomy, such as grooves, which may interfere with the correct seating of the surgical guide [19,20]. Therefore, in order to solve this problem in clinical practice, fine anatomical grooves are sealed with wax, or the fabricated surgical guides are given an offset during the design process [13,21,22,23]. Several studies have found that a higher offset results in higher accuracy of implant placement [23,24]. However, these studies have been insufficient, and their evaluation methods and range of offset values have differed [17,25,26]. Therefore, it is currently challenging to adopt a protocol while clinically designing the surgical guides.

The AM process is influenced by factors along with the different steps, involving acquisition and rendering of the 3D design data, technical [27] and material printing conditions [28], and the post-processing steps thereof [29]. With the rapidly evolving application of AM in the dental community, it is more essential than ever before to test and validate the accuracy of AM appliances and their efficacy [30].

The purpose of this study was to assess the accuracy of a surgical guide in terms of its internal fit and deviation of implant placement position. This was evaluated for two design variables: the effect of the groove sealing (GS) applied to the occlusal surface of the posterior teeth, and the range of offsets for the internal surface of the surgical guide. The null hypothesis of this study was that GS and the offset do not influence the internal fit of the 3D-printed surgical guide and the accuracy of implant placement.

## 2. Materials and Methods

### 2.1. Preparation of the Experimental Model

**Figure 1:**
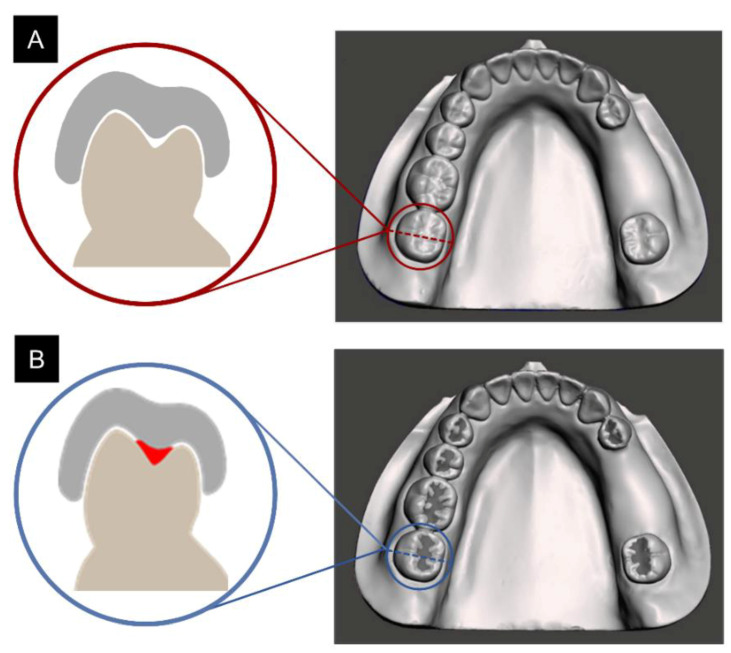
Experimental models. Samples were divided into two groups according to the sealing of the posterior groove: (**A**) original shape (OS) and (**B**) groove sealing (GS).

To prepare an experimental model, a fully dentate model (D85DP-500B.1, Nissin Dental, Kyoto, Japan) was scanned using a table-top scanner (Identica T500, Medit, Seoul, Korea). From the scanned CAD file, lower-right second premolars and first molars were removed using a 3D modeling program (Meshmixer 3.5, Autodesk, CA, USA). The simulated partially edentulous model was then exported as STL and 3D-printed formats (Form 3, Formlabs, MA, USA). The printed model was then replicated into a polyurethane model to ensure long-term dimensional stability. Briefly, a full-arch silicone impression of the 3D-printed model was made and poured with liquid polyurethane (Modralit 3K, Dreve Den-tamid, Unna, Germany), and allowed to polymerize at room temperature for one hour. The obtained polyurethane model was then used for all experiments, as shown in Figure 1.

### 2.2. Design and Production of Surgical Guides

The experimental polyurethane model prepared for the surgical guide design was scanned using a table-top scanner. Differences according to the shape of the occlusal surface were assessed by dividing the samples into a group in which the original anatomy of the posterior teeth was scanned in a tooth model (OS; original shape) and a group that was scanned after sealing the groove with paraffin wax (GS; groove sealing) (Figure 1).

**Figure 2:**
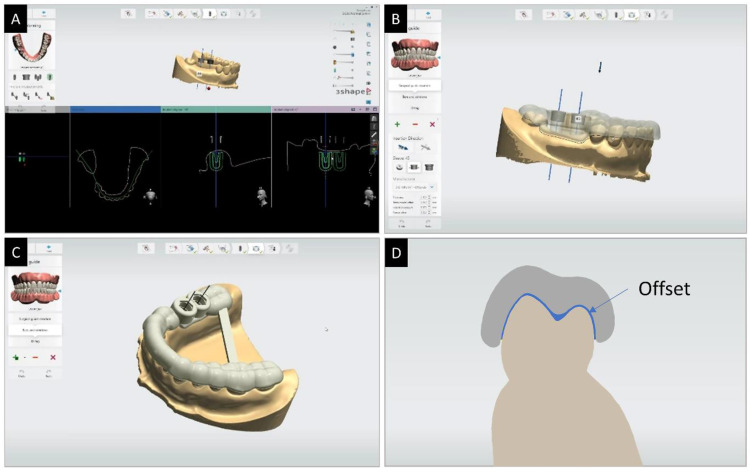
Implant placement plan and surgical guide design: (**A**) implant placement plan; (**B**) setting of the surgical guide (sleeve diameter of 5.7 mm, thickness of 2 mm, and offsets of 30 μm, 60 μm, and 100 μm); (**C**) bar addition and completed surgical guide design; and (**D**) uniform design offset on the inner surface of the surgical guide.

Two implants were planned and a full-arch supported surgical guide was designed using implant planning software (Implant Studio, 3Shape, Copenhagen, Denmark). The diameter of the sleeve was 5.7 mm, the thickness of the surgical guide was 2 mm, and the offsets from the tooth were 30 μm, 60 μm, and 100 μm. In addition, a bar connecting the left and right molars was added to prevent distortion that may occur during the 3D printing process of the surgical guide (Figure 2).

The design file of the surgical guide was transferred to 3D printing slicer software (Preform 3.14.0, Formlabs, MA, USA) and supports were attached to the outer surface of the surgical guide. The thickness of each printed layer was set to 50 μm. The 3D printer used to produce the surgical guide was a stereolithography apparatus (SLA) with an XY resolution of 25 μm, and a laser spot of 85 μm diameter, wavelength of 405 nm, and power of 250 mW. Printing was carried out with a maximum of 5 surgical guides per cycle with a 120° orientation and full-raft base with supports of touchpoint sizes of 0.80 mm. Photopolymer resin (Standard Grey Resin V4, Formlabs, MA, USA) for 3D printing was used as the printing material. Post-processing was followed by washing for 10 min in accordance with the manual provided by the manufacturer, and post-curing (Formcure, Formlabs, MA, USA) was performed for 30 min at 60 °C. Post-processing steps allowed the removal of excess resin, and following completion of the post-curing cycle, the supports were carefully trimmed manually using pliers.

### 2.3. Evaluation of Surgical Guide Internal Fit

The fit of the surgical guide was evaluated at the incisal and cusp tips of mandible teeth, except for the missing tooth, by measuring the difference in thickness of the silicone film obtained by the silicone replica method and analyzing in three dimensions. For evaluation of the implant placement position, 10 surgical guides per surface type (OS and GS) and for each offset value (30 μm, 60 μm, and 100 μm); a total of 60 surgical guides were printed.

The internal surface of the surgical guide was filled with a low-viscosity silicone indicator (Fit Checker Advanced, GC, Tokyo, Japan) and positioned over the experimental model. A force of 10 N was then applied to the external surface of the guide, and the silicone material was allowed to set. A shore hardness tester (LD-YJ, Vetus Industrial, Schiedam, The Netherlands) with a flat surface mounted on a jig was used to ensure that a uniform force was applied.

**Figure 3:**
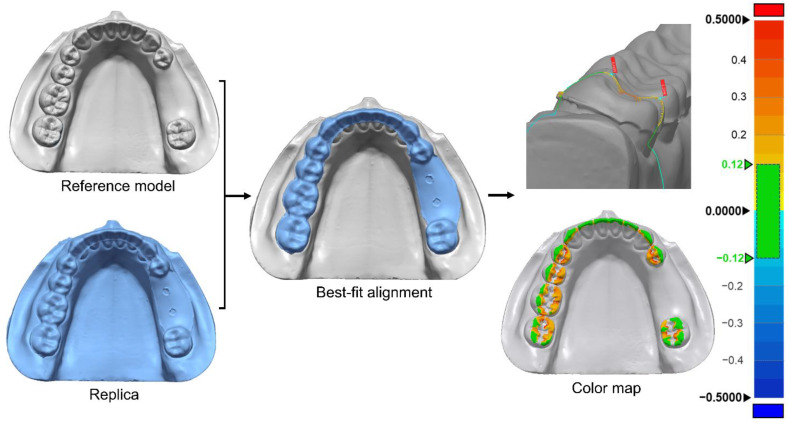
Process of the three-dimensional analysis of the internal fit of the surgical guide.

After the silicone material had set, the surgical guide was carefully removed such that the silicone film remained on the tooth model. The experimental polyurethane model covered with silicone film was scanned using a table-top scanner. This process was performed for each surgical guide. In order to determine the thickness of the silicone film, a model without the silicone film was also scanned and imported. Scanned data of the model with the silicone film were designated as the measured data, and those without the film were categorized as reference data for an evaluation performed using 3D inspection software (Geomagic Control X, 3D Systems, Rock Hill, SC, USA). To evaluate the deviation between the two groups, the coordinate values were first adjusted by overlapping the data using the alignment function of the software. Then, the meshes of the two datasets were overlapped as closely as possible using a best-fit algorithm. The root-mean-square error (RMSE) of the deviation of the thickness of the silicone film was calculated in the occlusal area using the software. A smaller RMSE value indicated a better internal surface fit. Color maps were produced covering an evaluation range of 500 μm, with a tolerance range of 120 μm (Figure 3).

### 2.4. Evaluation of Implant Placement Position

To evaluate the accuracy of implant placement, we analyzed the deviation between the positions of two planned and placed implants, at mandibular second premolar and first molar regions, using the surgical guide. For evaluation of the implant placement position, 10 surgical guides per surface type (OS and GS) and for each offset value (30 μm, 60 μm, and 100 μm); a total of 60 surgical guides were printed. This study used the research method proposed by Lim et al. [31] as a reference to reduce the error of the accumulated results during the actual implant placement process. This method was intended to evaluate the accuracy of the surgical guide itself by inserting the scan body into the surgical guide sleeve instead of placing the implant, and then simulating implant placement based on the position of the scan body. The scan body to be inserted into the sleeve was milled from titanium to match the diameter of the surgical guide sleeve, and the same scan-body design was registered in the library of CAD software (3Shape Dental System, 3Shape).

For the analysis, the surgical guides of each group were mounted on the experimental model, and then the scan body was inserted into the sleeve to obtain scan data using the table-top scanner. The acquired data were imported into the CAD software. In the same manner as in the general CAD process for an implant prosthesis, the scan body was replaced with the scan-body image registered in the library, the abutment of the implant prosthesis was created, and the file was exported. To analyze the difference in position, as for the reference data, a file was exported by creating an abutment based on the planned implant. Data for the two abutments were imported into 3D inspection software.

**Figure 4:**
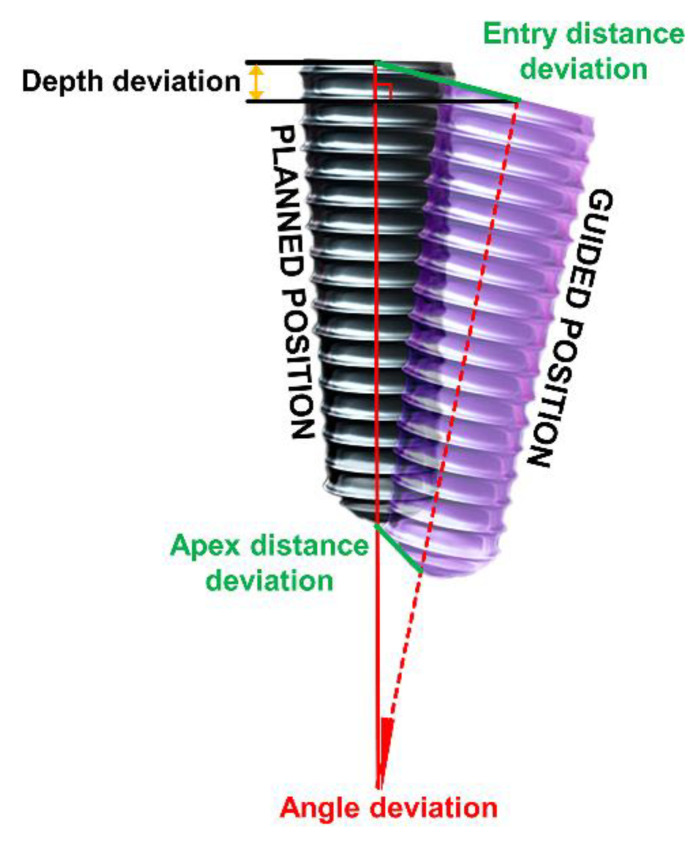
Measurement of the deviation between the planned position and the placed position by the surgical guide.

After aligning the coordinate values between the data by overlapping the reference centers using the initial alignment function of the software, the meshes of the two data-sets were overlapped as closely as possible using a best-fit algorithm. In order to reproduce the position of the implant fixture in each dataset, planes and lines were projected below the experimental model based on the position information of the abutment, and a fixture with a length of 10 mm was reproduced. The deviations at the entry and apex and in the depth and angle were evaluated for the two created data fixtures, with smaller deviations indicating better placement accuracy of the implant (Figure 4).

**Figure 5:**
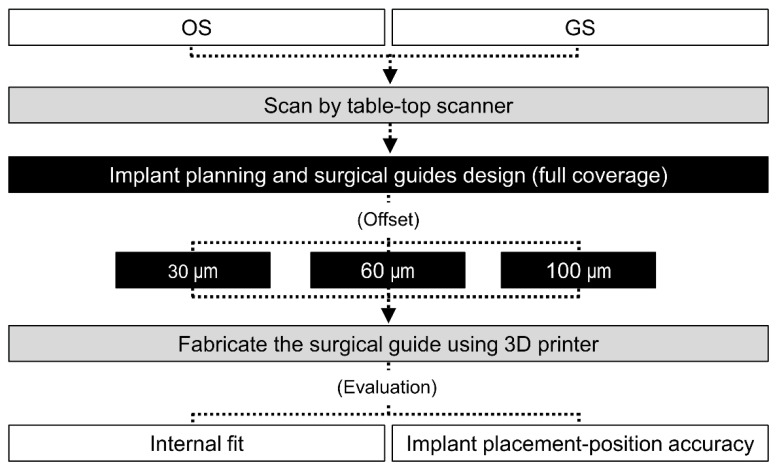
Flowchart of the overall experimental process of this study, showing the parameter groups and the evaluation methods. OS, original shape; and GS, groove sealing.

The entire experimental process of this study is shown in Figure 5. In order to minimize the bias of the results, the evaluator did not participate in the manufacturing process of the surgical guide. In addition, the order of measurement of the surgical guide was performed randomly, regardless of the order of its 3D printing.

### 2.5. Statistical Analysis

All statistical analyses were performed using SPSS software (version 25, IBM, NY, USA). Two-way ANOVA was performed to identify the factors affecting the accuracy of the surgical guide and the interaction according to surface shape and the offset. One-way ANOVA and Student’s *t*-test were used to determine the difference in the accuracy of the surgical guide for each factor, with a criterion of α < 0.05. For the post-test of each analysis, Tukey’s test was applied to evaluate any significant differences within each factor (also with α < 0.05). A post hoc power analysis (G*Power suite, 3.1.9.7, Düsseldorf, Germany) revealed that the sample size of 20 in each group for the three groups of 30 µm, 60 µm and 100 µm with a 5% error had a 98.11% power to detect the difference in means between the groups.

## 3. Results

### 3.1. Internal Fit of the Surgical Guide

**Figure 6:**
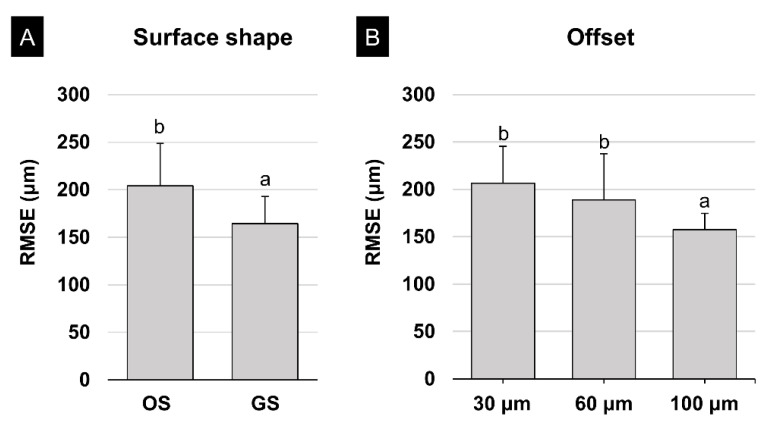
Results of two-way ANOVA for the fit of the surgical guide: (**A**) surface shape and (**B**) offset. Different lowercase letters indicate a significant difference (*p* < 0.05). Data are the mean and SD values. RMSE, root-mean-square error.

Two-way ANOVA was performed to verify the effects of surface shape and the offset on the fit of the surgical guide. It was found that surface shape (*F* = 41.011, *p* < 0.001) and the offset (*F* = 21.429, *p* < 0.001) affected the internal fit of the surgical guide. There was also a significant interaction effect between the two factors (*F* = 22.100, *p* < 0.001). The RMSE values were significantly lower for the GS than the OS (*p* < 0.001) (Figure 6A), and significantly lower for an offset of 100 μm (*p* < 0.001), with no significant difference between offsets of 30 μm and 60 μm (*p* = 0.071) (Figure 6B).

**Figure 7:**
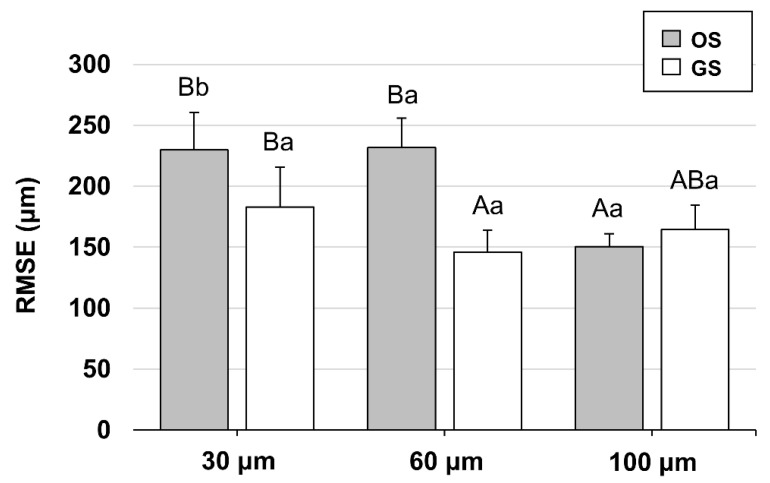
Results of one-way ANOVA for the fit of the surgical guide according to offset. Different uppercase letters indicate a significant difference between the offsets for each surface shape, and different lowercase letters indicate a significant difference between the shapes for each offset (*p* < 0.05). OS, original shape; and GS, groove sealing.

Figure 7 shows the differences in internal fit between and within groups for each factor. In the OS group, the RMSE values for offsets showed a significant difference (*p* < 0.001). The post hoc tests showed that the RMSE values were significantly lower for an offset of 100 μm than for offsets of 30 μm and 60 μm (*p* < 0.001) and did not differ significantly between 30 μm and 60 μm offsets (*p* = 0.987).

In the GS group, the RMSE values for offsets showed statistical differences (*p* = 0.009). The post hoc tests showed that the RMSE values were significantly lower for an offset of 60 μm than 30 μm (*p* = 0.07) but did not differ significantly from that for an offset of 100 μm (*p* = 0.304), or between offsets of 30 μm and 100 μm (*p* = 0.310).

The RMSE values were significantly lower in the GS group than the OS group for offsets of 30 μm (*p* = 0.004) and 60 μm (*p* < 0.001), but not for a 100 μm offset.

**Figure 8:**
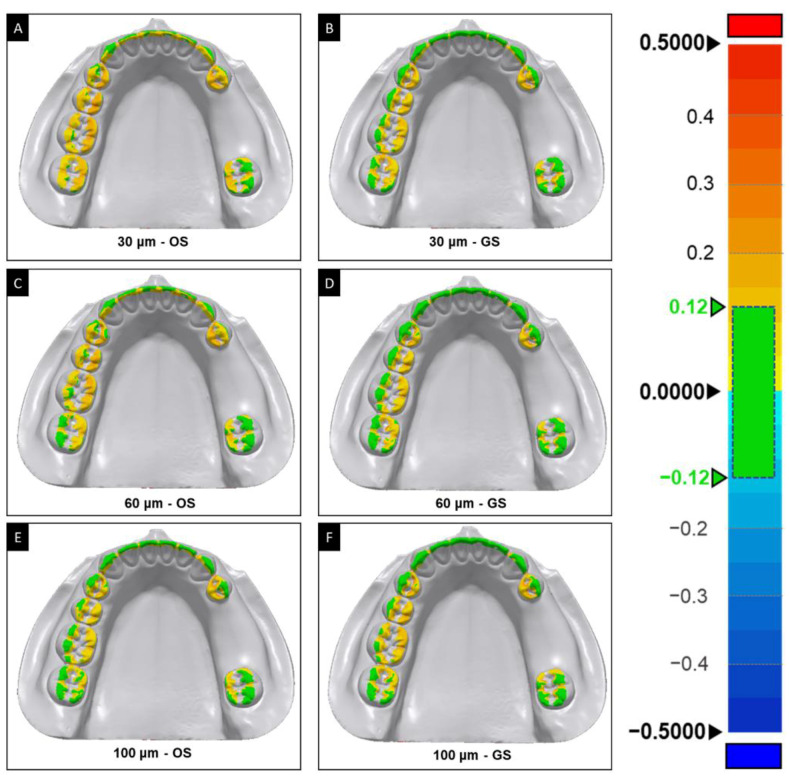
Color maps of the fit of the surgical guide according to surface shape and the offset using the same false-color scale as in Figure 3 for offsets of 30 μm (**A**,**B**), 60 μm (**C**,**D**), and 100 μm (**E**,**F**), and surface shapes of OS (**A**,**C**,**E**) and GS (**B**,**D**,**F**). OS, original shape; and GS, groove sealing.

Color maps of scan data superimposed on the silicone film in each group for the qualitative evaluation of the overall deviation are shown in Figure 7. Qualitative assessments revealed that there was a tendency for data values being more likely to fall within the tolerance range in the GS group than the OS group for different shapes of tooth surfaces. A smaller offset tended to make the color differences more distinct (Figure 8).

### 3.2. Evaluation of the Accuracy of the Implant Placement Position

**Figure 9:**
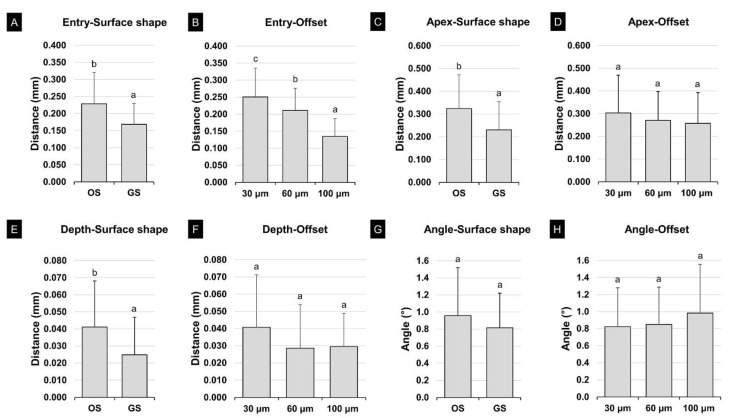
Results of two-way ANOVA for implant placement according to surface shape and the offset: (**A**) entry, GS; (**B**) entry, offset; (**C**) apex, GS; (**D**) apex, offset; (**E**) depth, GS; (**F**) depth, offset; (**G**) angle, GS; and (**H**) angle, offset. Different lowercase letters indicate a significant difference (*p* < 0.05). OS; original shape and GS; groove sealing.

Two-way ANOVA was performed for each measurement point to verify the effects of surface shape and the offset on the implant placement position. Although the angular deviation was statistically similar at the entry (*F* = 28.679, *p* < 0.001), apex (*F* = 13.779, *p* < 0.001), and depth (*F* = 14.060, *p* < 0.001), the deviations were significantly lower for the GS group than the OS group for the surface shape (Figure 9A,C,E,G). The entry (*F* = 36.789, *p* < 0.001) was only affected by the magnitude of the offset; each offset showed a significant difference (*p* < 0.05) (Figure 9B,D,F,H). In addition, there was a significant interaction effect between the surface shape and offset at depth (*F* = 3.149, *p* = 0.047).

**Figure 10:**
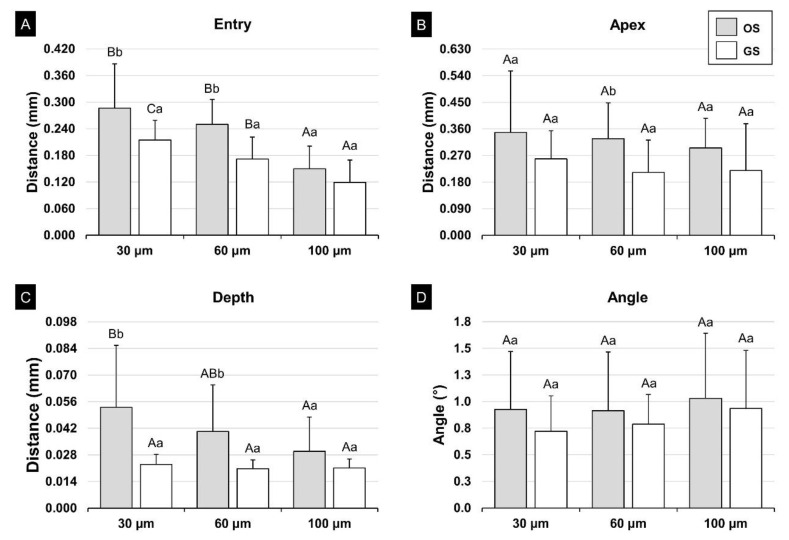
Results of one-way ANOVA for implant placement according to the offset: (**A**) entry, (**B**) apex, (**C**) depth, and (**D**) angle. Different uppercase letters indicate a significant difference between the offsets for each surface shape, and different lowercase letters indicate a significant difference between the shapes for each for each offset (*p* < 0.05). OS, original shape; and GS, groove sealing.

Figure 10 shows the results of one-way ANOVA for identifying deviations between and within the groups according to surface shape and the offset. In the OS group, the entry (*p* < 0.001) and depth (*p* = 0.023) were the significant differences for offset. In the results of post hoc tests, the deviation was significantly higher for an offset of 30 μm and 60 μm than for those of 100 μm at entry (*p* < 0.001). The depth was significantly higher for the offset of 30 μm than for one of 100 μm (*p* = 0.017), whereas it did not differ significantly for 60 µm offset on comparison with 30 μm (*p* = 0.271) and 100 μm (*p* = 0.415) offset, respectively. Therefore, and 100 μm (entry, *p* < 0.001; apex, *p* = 0.002; depth, *p* < 0.001) at entry. The angle was significantly higher for the offset of 30 μm than for one of 100 μm (*p* = 0.017), whereas it did not differ significantly for 60 µm offset on comparison with 30 μm and 100 μm offset, respectively. There were no significant differences in the deviation according to offset in the GS group except the entry (*p* < 0.001). In the results at entry of the post hoc test, the deviation was higher for an offset of 30 μm with significant differences among each offset (*p* < 0.05).

For an offset of 30 μm, the deviation was significantly lower for the GS group than the OS group for entry and depth points (entry, *p* = 0.007; depth, *p* < 0.009), and for an offset of 60 μm it was the same (entry, *p* < 0.001; depth, *p* < 0.002). Contrastingly, for an offset of 100 μm, there were no significant differences for any measurement points between the OS and GS groups.

## 4. Discussion

Three-dimensionally printed objects often shrink during the printing and post-curing processes, which can lead to the deformation of surgical guides manufactured using 3D printers with large variations along the printing axis [32], possibly affecting the final implant position [33]. Therefore, when manufacturing a surgical guide, it is necessary to utilize optimal parameters that provide stable seating. This study evaluated the effects of surface shape and the offset on the internal fit of 3D-printed surgical guides and its effect on the accuracy of implant placement. The accuracy was found to vary significantly with surface shape and the offset, and hence the null hypothesis of this study was rejected.

Printing errors in the 3D-printed model can arise from each link of the printing process and the parameters thereof. These include residual polymerization of the resin, effects of support structures, print resolution (*x* and *y* planes), layer thickness (*z* plane), and surface finishing [20]. The internal fit was significantly better for an offset of 100 μm than for offsets of 30 μm and 60 μm. This indicates that the appropriate offset can compensate for errors that may occur due to minor irregularities in the tooth surface on which a surgical guide is seated, thus improving the guide’s seating stability. In addition, for a surgical guide with GS, the internal fit was significantly better for offsets of 30 μm and 60 μm. When the offset is small, minor structures such as the occlusal groove can be a decisive factor in the incorrect seating of a surgical guide. Simplifying the occlusal anatomy using GS makes it possible to compensate for inaccurate seating caused by small offsets.

Ye et al. [23] evaluated the fit of a splint covering the occlusal surface that was designed with various settings. They found that the internal deviation decreased as the offset increased when comparing between the group without offset and groups with offsets from 50 to 200 μm, which was consistent with the present findings. Their study also found that while the accuracy was better for an offset of 200 μm than for one of 100 μm, the higher offset resulted in larger buccolingual movement. In other words, excessive space between the surgical guide and teeth tends to reduce the overall stability of the guide. Therefore, choosing an appropriate offset is a decisive factor towards the stability of the surgical guide and accurate implant placement.

The present study also analyzed the effect of the surgical guide’s surface shape and the offset on the accuracy of implant placement. The error of the implant position was highest for an offset of 30 μm, with the implant placed vertically shallower compared to the planned depth. The entry showed similar results. The range of the offsets investigated in the present study (30–100 µm) differed somewhat from those compared in the study of Neumeister et al. [24] (100–300 µm). However, the tendency of increasing vertical deviation of implant placement with a decreasing offset was concordant with our study. This can be attributed to the incorrect seating of the surgical guide leading to the occurrence of vertical and horizontal errors. The fixture of the implant was connected to the abutment; therefore, the depth is related to the effect on the peri-implant tissue, and the inaccurate entry can lead to inadequate implant prostheses [13,34]

Additionally, we evaluated the internal fit and the resultant accuracy of the implant placement position influenced by the surface shape and offset. The accuracy of both the internal fit and implant placement showed clear improvements following minor changes in the anatomy of the occlusal surface through GS along with a 30 μm offset.

When designing the surgical guide with a metal sleeve, a consistent tactile feedback during the implant placement was considered as an advantage; however, in the surgical guide (with and without a metallic sleeve), improved control of the drill movement could be achieved by design modification in the length of the sleeve [35]. In addition, Tallarico et al. [36] found that the accuracy of the surgical guide designed with open hole was lower compared to the close hole design, which could again be attributed to better drill support achieved in the closed hole design. Therefore, the authors recommended the use of a sleeve with an open hole only in cases with limited access in the posterior areas [36]. Notwithstanding the above, from the results of the present study, it could be inferred that the application of groove sealing and offset in the surgical guide design process could also significantly influence the stability of a surgical guide. This would especially be more applicable in cases of open hole-type designs, which warrant a superior stability to overcome limitations in drill support.

The conventional way to evaluate the accuracy of a surgical guide is by comparing the positions of planned and placed implants based on CBCT and digital data. Ma et al. [37] compared the two traditionally used evaluation methods for 3D-printed surgical guides and demonstrated that evaluations based on CBCT were significantly less accurate than those based on digital data. Their analyses of digital data found that the deviation was 0.82 ± 0.44 mm in the coronal area, 1.19 ± 0.46 mm at the apex, and −0.03 ± 0.65 mm at depth, with an angular deviation of 2.43° ± 1.13°. These results differ from those of the present study, in which a good accuracy was obtained when using a surgical guide with an offset of 100 μm, with a deviation of 0.150 ± 0.051 mm at the entry, 0.296 ± 0.100 mm at the apex, 0.030 ± 0.018 mm at depth, and an angle deviation of 1.030 ± 0.610°. The difference with our results can be better interpreted while considering the technical variability between the two methods.

An evaluation method that compares the position of implant placement by placing implant fixtures directly in a patient’s oral cavity or dental model carries an inherent tendency for bias due to the surgical procedure being affected by the surgical guide, operator’s skill, or due to the presence of an edentulous area [38,39]. Furthermore, evaluations of implant positions using CBCT data may be limited by deteriorations in the quality of CBCT images due to patient movement errors and metal artifacts [38,40]. Therefore, in this study, instead of directly placing an implant in the model to evaluate the position of the surgical guide, a scan body was inserted into the sleeve on the surgical guide and scanned, and the position of the implant was calculated using library data.

The precise fit of the scan body and sleeve can be an important factor influencing the accurate detection of the position of the surgical guide. In this regard, it has been reported that a scan body inserted into the sleeve of the surgical guide has the same level of reproducibility as a scan body connected directly to the implant fixture. The method that was utilized to evaluate implant position in this study may be more convenient because it omits the surgical procedure in in vitro studies. Moreover, in clinical practice, it has the advantage of being able to precheck and correct the accumulated errors by evaluating the expected implant position for the surgical guide to be used prior to actually performing implant surgery [31]. Therefore, the lower deviations in implant position observed in our study could be attributed to the above modifications in the scanning and evaluation of implant position.

However, this study performed nonclinical investigations and has the limitation that it does not reflect the oral environment. Therefore, there may be differences in the results obtained in clinical studies in which complex anatomical factors play a role. Especially, this study objective had a focus limited to the factors in design and planning of a 3D-printed surgical guides such as groove sealing and adjustment of the offset amount; therefore, for ease of obtaining an accurate and reproducible scan, the choice of 3D printing photoactive resin was a grey methacrylate-based resin that does not have translucent properties. Although the material is not indicated for direct intraoral application, it can be expected that the results will be minimally affected by the choice of material owing to the similar chemical nature to the commonly used dental methacrylate resin. However, because AM appliances are known to be affected by the changes in material type, a cautious interpretation of the results from this study should be considered. Future studies performing comparisons using similar methods in this study are required to obtain clinically relevant data with medical-grade materials.

To improve the accuracy of the implant placement, groove sealing and offset of the occlusal surface must be carefully considered for the inner surface during surgical guide fabrication. The simultaneous optimization of both factors might be challenging; however, with the results of the present study, it can be suggested that customizing either factor may contribute to markedly improving the implant placement accuracy. Surface shape is likely to be more helpful in patients with irregular tooth surfaces to improve the accuracy of implant surgery. Although the 3D printing of surgical guides is a multifactorial procedure, implementing a standard protocol with design factors, such as surface shape and the offset, will help to improve clinical outcomes with a higher predictive accuracy.

## 5. Conclusions

The accuracy and the fit of a 3D-printed (SLA) surgical guide can be significantly improved by factoring occlusal groove sealing and offset, prior to the design and manufacturing.

## Data Availability

The data presented in this study are available on request from the corresponding author.
